# Assessing the greenhouse gas emissions of Brazilian soybean biodiesel production

**DOI:** 10.1371/journal.pone.0176948

**Published:** 2017-05-11

**Authors:** Carlos Eduardo Pellegrino Cerri, Xin You, Maurício Roberto Cherubin, Cindy Silva Moreira, Guilherme Silva Raucci, Bruno de Almeida Castigioni, Priscila Aparecida Alves, Domingos Guilherme Pellegrino Cerri, Francisco Fujita de Castro Mello, Carlos Clemente Cerri

**Affiliations:** 1 University of São Paulo, “Luiz de Queiroz” College of Agriculture, Department of Soil Science, 11 Pádua Dias Avenue, Piracicaba, SP, Brazil; 2 University of Hohenheim, Faculty of Agricultural Sciences, Institute of Soil Science and Land Evaluation (310), Stuttgart, Germany; 3 University of São Paulo, Center for Nuclear Energy in Agriculture, 303 Centenário Avenue, Piracicaba, SP, Brazil; 4 Brazilian Vegetable Oil Industries Association, 3707 Vereador José Diniz Avenue, São Paulo, SP, Brazil; 5 Espaço Eco Foundation, 230 Ribeirão do Soldado Road, São Bernardo do Campo, SP, Brazil; 6 DeltaCO2 –Sustentabilidade Ambiental, 600 Cezira Giovanoni Moretti Street, Piracicaba, SP, Brazil; 7 Department of Development of Production Chains and Sustainable Production at the Ministry of Agriculture, Livestock and Food Supply, Esplanada dos Ministérios, Bl. D. Anexo 1ºandar-Ala B- Sl. 137, Brasília, Brazil; International Centre for Genetic Engineering and Biotechnology, INDIA

## Abstract

Soybean biodiesel (B100) has been playing an important role in Brazilian energy matrix towards the national bio-based economy. Greenhouse gas (GHG) emissions is the most widely used indicator for assessing the environmental sustainability of biodiesels and received particular attention among decision makers in business and politics, as well as consumers. Former studies have been mainly focused on the GHG emissions from the soybean cultivation, excluding other stages of the biodiesel production. Here, we present a holistic view of the total GHG emissions in four life cycle stages for soybean biodiesel. The aim of this study was to assess the GHG emissions of Brazilian soybean biodiesel production system with an integrated life cycle approach of four stages: agriculture, extraction, production and distribution. Allocation of mass and energy was applied and special attention was paid to the integrated and non-integrated industrial production chain. The results indicated that the largest source of GHG emissions, among four life cycle stages, is the agricultural stage (42–51%) for B100 produced in integrated systems and the production stage (46–52%) for B100 produced in non-integrated systems. Integration of industrial units resulted in significant reduction in life cycle GHG emissions. *Without the consideration of LUC and assuming biogenic CO*_*2*_ *emissions is carbon neutral in our study*, the calculated life cycle GHG emissions for domestic soybean biodiesel varied from 23.1 to 25.8 gCO_2_eq. MJ^-1^ B100 and those for soybean biodiesel exported to EU ranged from 26.5 to 29.2 gCO_2_eq. MJ^-1^ B100, which represent reductions by 65% up to 72% (depending on the delivery route) of GHG emissions compared with the EU benchmark for diesel fuel. Our findings from a life cycle perspective contributed to identify the major GHG sources in Brazilian soybean biodiesel production system and they can be used to guide mitigation priority for policy and decision-making. Projected scenarios in this study would be taken as references for accounting the environmental sustainability of soybean biodiesel within a domestic and global level.

## Introduction

Environmental sustainability has been a major concern of bioenergy industry in the last decade and a number of indicators have progressively emerged in scientific literature [1–5]. Greenhouse gas (GHG) emission is one of the primary indicators associated to global climate changes and plays a key role within various sustainability analysis approaches [6,7]. The United Nations (UN) and several regional authorities have set sustainability criteria regarding GHG emissions for energy sources and biodiesel is one of the major subjected products [8–10].

To meet the growing global demand of biofuels, biodiesel has been an emerging alternative to fossil diesel fuel to guarantee environmental sustainability, especially in Brazil [11]. Since the Brazilian National Program of Production and Use of Biodiesel (PNPB) was launched in 2004, its actions have further insured the introduction of biofuels into Brazilian energy matrix, including the mandatory use of B5 (*i*.*e*., 5% biodiesel blended with 95% petroleum diesel) since 2010 [12] and the mandatory use of B8 from 2017 [13]. In the last decade (2005–2015) the Brazilian biodiesel production increased rapidly from 0.736 million liters to 3.9 billion liters [14], making Brazil one of the leading global producers of biodiesel [15]. Among all the inputs, soybean is the predominant feedstock for biodiesel production, with a contribution of 77% to the total biodiesel production in 2014 in Brazil [14]. Brazilian soybean also plays an important role globally. About 23% of the biodiesel processed in Brazil has been exported mainly to Europe and the Brazilian soybean-derived biodiesel prevails vastly in Spain, France, Italy and Portugal [16, 17].

In Brazil, soybean cultivation was originally concentrated in the southern region. However, in the last decades it has been extended through the Cerrado and Amazon regions especially in the state of Mato Grosso, which accounts for about 1/3 of the total soybean production in Brazil [18]. The new road (BR-163) connecting Cuiabá (Mato Grosso state) to Santarém (Pará state) and the improvements in the port infrastructures in Santarém are boosting even more the cultivation of soybean in Mato Grosso, making it the largest producing region of raw material for the Brazilian soybean biodiesel industry [19].

Castanheira et al. [20] conducted a life cycle assessment (LCA) to assess the environmental impacts of three Brazil-based soybean biodiesels in Europe. Although this paper provided an important holistic view of the soybean biodiesel production chain, the use of aggregate data (e.g., national average, public database, etc.) reduces the level of details, tends to amplify uncertainty and decreases the reliability of the results. Thus, data quality becomes one of the obstacles to LCA [21–24]. Despite the systematic view in LCA, GHG emissions, often contextually referred to with the term carbon footprint, has a much broader appeal and is widely used as an indicator of environmental sustainability [25–29]. This indicator has raised environmental awareness among decision makers in business and politics, as well as the particular attention from consumers. For better understanding the environmental impacts of Brazilian soybean biodiesel, a life cycle GHG emission assessment would provide a higher resolution of details and suggest more dependable ways for policy making.

Several studies have reported a wide range of GHG emissions from the agricultural production stage of Brazilian soybean, some even considered the part of the transportation and biodiesel production stages [20, 30–36]. However, the results vary significantly due to different normative and methodological choices in the assessments (Table 1). Raucci et al. [35] performed 114 individual evaluations of the GHG emissions of soybean cultivation in Brazil and suggested further systematic study for Brazilian soybean-derived products (e.g., soy meal, soybean oil, biodiesel and glycerin). Among all these products, soybean biodiesel has a high international demand and its environmental impact receives extensive attention by the EU, USA and UN [8–10]. Similar assessments have been conducted for soybean biodiesel [37,38] and biodiesels derived from other feedstock, such as rapeseed, jatropha and palm in EU [39]. All these studies have shown significant reduction in GHG emission compared to fossil fuels.

**Table 1:** Previous LCA emission studies of Brazilian soybean (-derived) products.

Functional Unit (FU)	Year	Tool	Stage*	kg CO_2_ eq/ FU	Comments	Reference
1000kg of feed	2009	LCA^1^	Agriculture	391	Data from public databases	[30]
1000 kg of feed	2012	LCA^1^	Agriculture	513–751	Ecological footprint vs. LCA methodologies; data from public databases	[33]
1000 kg of soybeans	2010	LCA^1^	Agriculture, Distribution	510–959	GHG emissions; Central West Brazil to Europe; data from public databases; **LUC included**	[32]
1 kg of soybean	2013	LCA^1^	Agriculture, Distribution	0.10–17.8	GHG emissions; data from national reports or other studies; **LUC included**	[34]
1 kg of soybean	2015	SM^5^	Agriculture	0.102–0.347	GHG emissions; primary data from farms	[35]
1 kg of soybean	2016	SM^5^	Agriculture	0.352–3.41	GHG emissions; primary and secondary data; **LUC included**	[36]
1 liter of biodiesel	2010	EA^2^, EEA^3^, MFA^4^	Agriculture	0.860 (only CO_2_)	Data from field work scientific literatures	[31]
1 MJ of energy	2015	LCA^1^	Life Cycle	0.132–0.137	Energy allocation; ReCiPe and USETox; **LUC included**	[20]

(* life cycle was defined in this study into four stages: agriculture, extraction, production and distribution

^1^LCA—life cycle assessment

^2^EA—Emergy Accounting

^3^ EEA–Embodied Energy Analysis

^4^ MFA–Material Flow Accounting

^5^SM–Spreadsheet-based modeling).

Considering Brazil-contextualized soybean biodiesel production chain and its various distribution scenarios, we hypothesized that the production of Brazilian soybean biodiesel is a sustainable way to mitigate GHG emissions as an alternative biofuel to fossil fuels, while technologically improved production systems would further enhance its environmental performance. Therefore, the main objective of this study was to assess GHG emissions of Brazilian soybean biodiesel with the first-hand data obtained from Brazilian biodiesel industry and associations. A comprehensive evaluation of the four life cycle stages was performed and several aspects of the soybean biodiesel production chain, including the integrated production system, the different energy efficiency of refineries and the various transportation routes were taken into account.

## Materials and methods

The sources of data used in the calculation of GHG emission were obtained from reference projects running by APROSOJA (Mato Grosso State Soybean & Corn Producers Association), ABIOVE (Brazilian Vegetable Oil Industry Association) and UBRABIO (Brazilian Biodiesel and Biokerosene Union). From the reference project, we used data from 55 farm members of APROSOJA, not only spatially distributed but also technologically representative from the largest soybean producer state in Brazil, 4 industrial members of ABIOVE and 1 industrial member of UBRABIO. The reference project collected the whole dataset with authorization from farmer-owned or company’s managers. This study did not involve endangered or protected species, so no formal permissions were needed from regulatory agencies. The main concern of this study is the environmental sustainability, thus, socio-economic aspects were not considered in this study. Carbon dioxide (CO_2_), nitrous oxide (N_2_O) and methane (CH_4_) are the three chemical contributors taken into account for GHG emission calculation and were expressed with CO_2_ equivalent (CO_2_ eq.) considering its global warming potential: CH_4_, kg × 25+ N_2_O, kg × 298+ CO_2_, kg [8].

### System description

**Fig 1:**
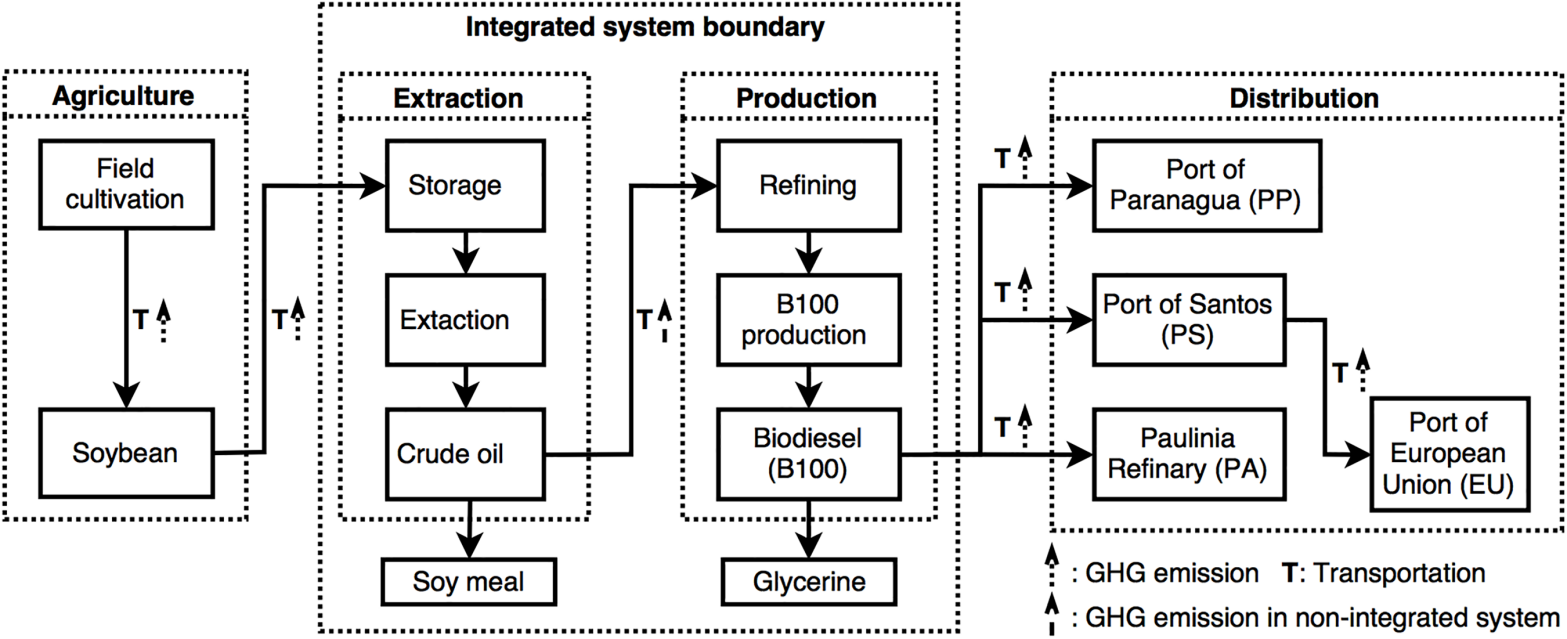
Life cycle of soybean biodiesel produced in Brazil, highlighting GHG emission sources within the four stages: agriculture, extraction, biodiesel production and distribution.

The GHG assessment included data from four life cycle stages in the soybean biodiesel production chain (Fig 1). Specific to extraction and production stages, two different industrial configurations were considered. In a non-integrated system, soy oil extraction and biodiesel production are performed in different industrial plants, while an integrated system combines, in the same industrial unit plant, both stages in a consecutive production chain (Fig 1).

#### 1^st^ Stage—Agriculture

This stage comprised field GHG data from 55 commercial farms located in Mato Grosso (MT) state, central-west Brazil, accounting for 180,000 ha of soybean cultivation area. The GHG-emission inventories were performed in 2007/2008 (36 farms), 2008/2009 (32 farms) and 2009/2010 (46 farms) growing seasons, providing a total of 114 evaluated sites. We selected the farmers with the assistance of the largest soybean producer association (APROSOJA) that acts within studied region, in order to comprise farms with different production scales and scattered throughout the state. Direct-GHG emissions in soybean production stage (*i*.*e*., from the cradle to farm gate) included operations such as soil tillage, liming, sowing, fertilizer application, crop protection and harvest. Furthermore, indirect-GHG emissions from the production and transportation to farm of agricultural inputs (e.g., fuels, fertilizers, lime, pesticides, seeds and electricity) (Table 2) and decomposition of soybean crop residues were also taken into account. A detailed description about the study sites, GHG data and main inputs used in the soybean production was previously provided by Raucci et al. [35].

**Table 2:** Main inputs and yield per hectare of soybean in Mato Grosso state, Brazil (growing season of 2007/08, 2008/09 and 2009/10).

Growing season	2007/08		2008/09		2009/10	
	Average	Range	Average	Range	Average	Range
***Inputs***						
Diesel oil (L)	30	15.7–45.8	36	22.1–58.0	27	20.0–41.9
Fertilizers (kg)						
N	8	0.2–16.1	5	2.7–8.3	7	2.0–13.4
P_2_O_5_	84	64.4–161.1	82	49.2–131.6	78	37.3–141.8
K_2_O	90	52.6–145.1	89	57.2–131.6	83	37.3–125.0
Limestone (kg)	333	102.0–610.8	489	178.4–722.9	439	101.5–1319.0
Seeds (kg)	46	30.6–67.3	53	36.0–88.6	48	31.2–94.5
Electricity (kWh)	18	1.8–104.0	23	3.9–72.4	28	3.4–136.6
Pesticides (kg)						
Herbicides	3.85	0.12–10.91	3.94	0.22–7.31	5.85	0.18–11.29
Fungicides	0.95	0.03–2.37	1.11	0.17–2.68	1.40	0.02–3.76
Insecticides	1.61	0.04–8.13	2.00	0.18–5.31	1.83	0.04–6.45
***Output***						
Soybean	3316	2783–3805	3157	2331–3670	3129	2413–3672

Although land-use change (LUC) is an important source of GHG emission and has been added in other LCA studies, we did not include it because we were unable to gather the necessary data to adequately address such complex issue.

#### 2^nd^ stage–extraction

This stage comprised GHG emissions from storage of soybean grains and oil extraction into factories. The GHG-emission inventories were performed based on data provided by four companies affiliated to ABIOVE, which are the primary soybean-processing companies acting in the region, and are among the largest soybean-processing companies in the world. The main sources of GHG emissions evaluated included stationary combustion in generators, boilers and silos; electricity consumed in factories and silos; and indirect emissions from the production and transportation of industrial inputs and fuels.

#### 3^rd^ stage—Biodiesel production

This stage included GHG-emission inventories of the refining of crude soybean oil and industrial biodiesel production based on data from two of the largest biodiesel-producing companies acting in Brazil. As mentioned above, we considered two configurations of the industrial plants for producing biodiesel, integrated and non-integrated. For both scenarios, the main sources of GHG emissions came from stationary combustion in generators and boilers; electricity consumed in factories; indirect-GHG emissions from the production and transportation of industrial inputs (e.g., hexane, methylates, methanol, nitrogen among others) and fuels.

#### 4^th^ stage–distribution

**Fig 2:**
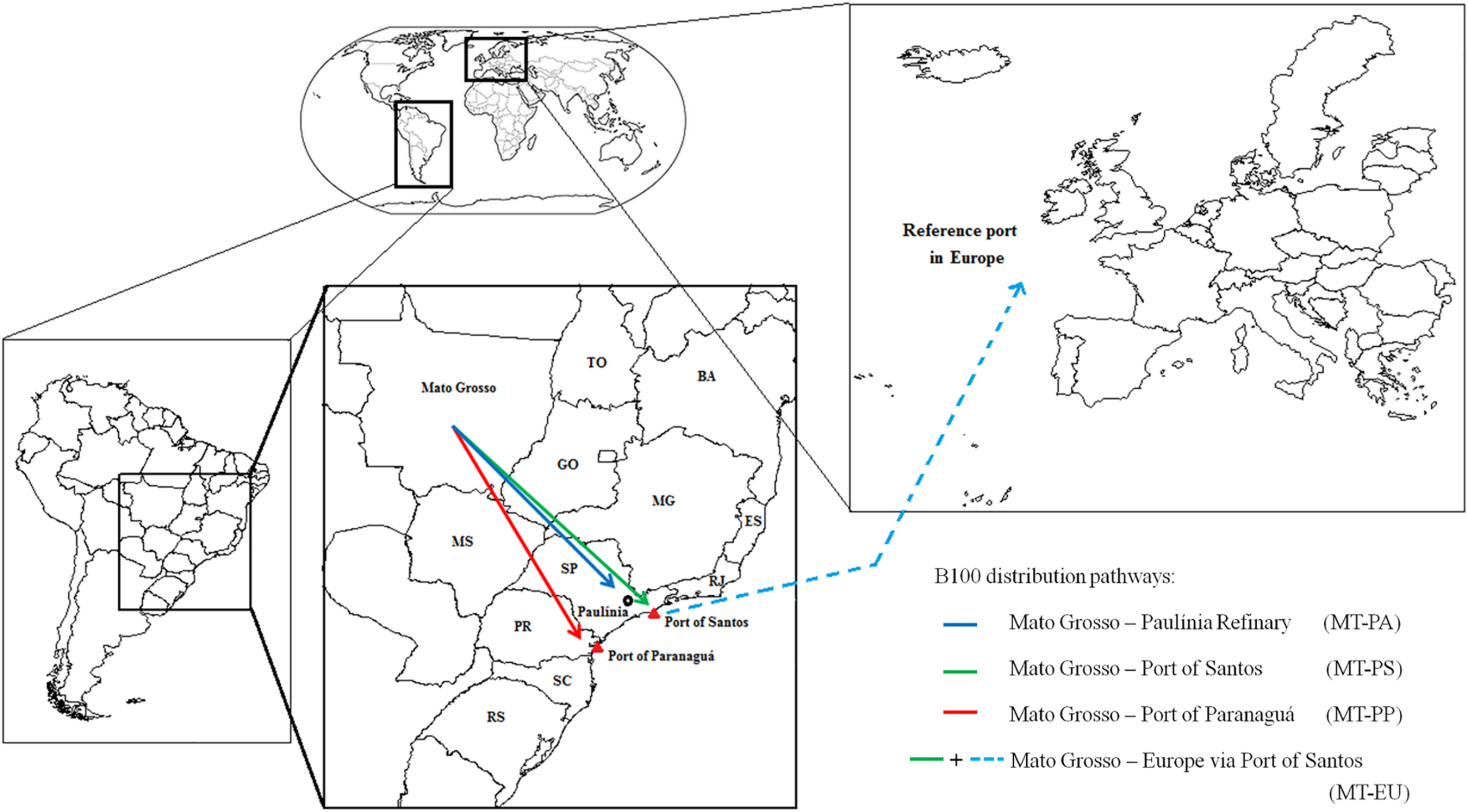
Schematic representation of the four pathways for distribution of final B100.

The final stage comprises four different delivery routes of biodiesel produced in Mato Grosso (MT) state, central-west Brazil. GHG-emission inventory of fuel consumption for the vehicles used in this transportation was obtained from Brazil’s Road Transport Association. We calculated the GHG emissions of final B100 delivered to four destinations (Fig 2): i) MT to Paulínia Refinery (Paulínia city, São Paulo state; pathway: MT-PA, about 1,200 km), where this large soybean oil refinery produces and assembles soybean biodiesel for further domestic distribution; ii) MT to Port of Santos (Santos city, São Paulo state; pathway: MT-PS, about 1,400 km), which is the largest port of the Brazil, and consequently, primary flow pathway for exportation of Brazilian soybean biodiesel; iii) MT to Port of Paranaguá (Paranaguá city, Paraná state: pathway: MT-PP, about 1,600 km), which is the second largest port of the Brazil, thus an important flow pathway for exportation of Brazilian soybean biodiesel; and iv) MT to a reference port in Europe (pathway: MT-EU, reference port adopted by the European Directive [9]), which considers a distance of 5,500 nautical miles (*i*.*e*., 10,186 km) between Brazil and the European Union port [40].

### Reference period

The reference period for this study was from June 2008 to July 2009. This period was defined as the base year for the GHG-emission inventories, in which all data were collected. The selected reference period is representative of the regular conditions of soybean biodiesel production system in Brazil. Nevertheless, GHG-emission data from soybean production stage consisting of an average of 2007/08, 2009/10 and 2010/11 crop seasons. We considered three consecutive years for the agricultural stage to encompass potential variations on field management practices (e.g., liming, application of organic manure, etc), making these data more consistent and representative.

### Calculation of GHG emissions

The GHG-emission calculations were performed individually for each farm in the agricultural stage and for each industrial unit in the extraction and production stages as well as for each pathway in the distribution stage. Specific to the extraction and production stages, a general consideration of the difference between integrated and non-integrated production chain was adopted regardless of the individual efficiency of each working unit. The international standards ISO 14040 and ISO 14044 were used to guide the allocation criteria. Whenever several alternative allocation procedures seem applicable, a sensitive analysis shall be conducted to illustrate the consequences of the departure from the selected approach [7]. Therefore, two allocation procedures were applied in this study based on mass and energy. Emission factors from multiple sources were adopted [8, 9, 41–45]. Conversion factors used are summarized in Table 3.

**Table 3:** Summary of conversion factors used in this study.

Product	Allocation approach			
	Mass		Energy	
	Factor (%) for integrated plant	Factor (%) for non-integrated plant	Factor (%)	Lower heating value (MJ kg^-1^)
Biodiesel	98	90	96	
Glycerin	2	10	6	
Oil	20		36	39.43
Meal	80		64	19.39

Mass allocation was chosen as the main allocation rule for this study, which is commonly used in LCA studies, because it is easy to be applied and provides reasonable results [46]. Accompanying energy allocation was used in accordance with the EU Directive on Renewable Energy Sources in the discussion as well as to test the sensibility of the results. Allocation of economic value was not applied in this study, not only because it is not the scope of this study but also because the economic value of soy oil and soy meal are volatile, requiring data to be updated frequently.

The life cycle GHG emission analyses of final B100 product based on four different pathways (MT-PA, MT-PS, MT-PP, MT-EU) were performed and the environmental implications are discussed based on the EU Directive on Renewable Energy Source (2009/28/EC). Finally, a comparison of life cycle GHG emissions of domestic and export B100, and the performances of integrated and non-integrated system were also presented.

For more details about GHG emission calculations see S1 Appendix.

## Results and discussion

### GHG emissions from each life cycle stage

In the agricultural stage, GHG emissions averaged 316 g CO_2_eq. kg^-1^ of soybean, which is one of the intermediate products in the life cycle of B100. Greenhouse gas emission values found in this study fell within a range consistent to other studies that used similar scope and boundaries in this stage [9, 34, 36, 45, 47, 48]. The field soybean cultivation shows large variability of GHG emissions associated to geographic location, soil type and use of different management practices and inputs [34–36]. In addition, although GHG assessments follow international guidelines (e.g., ISO, IPCC), each study has its own assumptions, which may modify the final results. For instance, when C debit from land use change is included in the inventory scope, GHG emissions might change abruptly [34, 36, 45, 48]. Therefore, this variety of results among GHG inventories reinforces the uncertainty to compare data from different regions or to assume some GHG estimate as a reference (default) for larger scale studies.

Crude soy oil and soy meal are produced in the extraction stage (Fig 1), with a mass allocation of 20% for the crude oil and 80% for the soy meal [38]. Soy oil processed by different companies, applying integrated and non-integrated industrial units, yielded GHG emissions ranging from 42 to 55 gCO_2_eq. kg^-1^ B100. Separate calculations of life cycle GHG emissions for the soy oil and soy meal were performed (Table 4). The GHG emissions for soy meal ranged from 641 to 710 gCO_2_eq. kg^-1^ B100, whereas emissions for soy oil extraction, which is prepared for biodiesel production, ranged from 649 to 709 gCO_2_eq. kg^-1^ B100. Finally, calculated GHG emissions for biodiesel production stage ranged from 168 to 510 gCO_2_eq. kg^-1^ B100. The variation in result is mainly due to the consideration of integrated and non-integrated industrial units for soy oil extraction, crude oil refining and biodiesel production. Generally, integrated plant has a much higher B100 production efficiency [*i*.*e*., 98% of B100 efficiency in integrated plant compared to 90% of non-integrated plant (Table 3)] and, consequently lower GHG emissions in by-products (Table 4). Thus, our results indicate that integrated plants have a more sustainable production concept, which relieves GHG emissions for B100 and its by-products (*i*.*e*., soy meal and soy oil).

**Table 4:** Life cycle GHG emission of product/by-product in the production chain of B100.

Products/ By-products	GHG emission (gCO_2_eq. kg^-1^ B100)		GHG emission (gCO_2_eq. MJ^-1^ B100)	
	Integrated	Non-integrated	Integrated	Non-integrated
Soybean	316	316	8.0	8.0
Soy meal	641	701	16.2	17.7
Soy oil	649	709	16.4	17.9

In the following distribution stage, the GHG emissions of the final delivered B100 varied in accordance with the destinations. For Paulínia Refinary (PA), Port of Santos (PS) and Port of Paranaguá (PP), the GHG emissions ranged from 89 to 99, from 101 to 112 and from 116 to 126 gCO_2_eq. kg^-1^ B100, respectively. Based on biodiesel delivered in PS, the GHG emissions of exported B100 to Port of European Union significantly increased, ranging from 215 to 226 gCO_2_eq. kg^-1^ B100.

### Life cycle GHG emissions of final B100

For the four distribution routes, a life cycle GHG emissions of B100 was calculated based on allocation of mass and energy (Table 5). Among the scenario of domestic distribution, B100 delivered to PA has the lowest GHG emissions (*i*.*e*., from 615 to 980 gCO_2_eq. kg^-1^ B100; from 23.1 to 25.8 gCO_2_eq. MJ^-1^ B100) and B100 delivered to PP has the highest one (*i*.*e*., from 755 to 1107 gCO_2_eq. kg^-1^ B100; from 26.5 to 29.2 gCO_2_eq. MJ^-1^ B100). For the scenario of exported B100 based on PS to the port in European Union, GHG emissions have further increased, reaching values that ranged from 775 to 1107 gCO_2_eq. kg^-1^ B100; 26.5 to 29.2 gCO_2_eq. MJ^-1^ B100. The uncertainties found can be associated mainly to the differences between integrated and non-integrated production system, the efficiency of chosen refinery for each product and the difference in final destination.

**Table 5:** Life cycle GHG emission of B100 based on four different transportation routes.

Pathway	Life cycle GHG emission			
	gCO_2_eq. kg^-1^ B100		gCO_2_eq. MJ^-1^ B100	
	Integrated	Non-integrated	Integrated	Non-integrated
MT-PA	615	980	23.1	25.8
MT-PS	627	993	23.4	26.1
MT-PP	642	1007	23.8	26.5
MT-EU	755	1107	26.5	29.2

A few studies around world have assessed the life cycle GHG emissions of soybean biodiesel [20, 37, 38]. However, it is difficult to make comparison with our findings, since each study uses different scenarios, approaches and units. For other biodiesel feedstocks, Uusitalo et al. [39] reported life cycle GHG emissions of approximately 50 gCO_2_eq. MJ^-1^ for rapeseed oil, 30 gCO_2_eq. MJ^-1^ for jatropha oil and 20 gCO_2_eq. MJ^-1^ for palm oil. Therefore, irrespective of land use change, our results are consistent with these values and even showed a slight advantage when comparing to rapeseed oil. Based on these difficulties to compare literature data, it is worth highlighting that standardized frameworks (*i*.*e*., scenario, approach, unit, etc.) regarding to life cycle GHG emission assessment of biodiesel should be a high priority for a better transition from scientific work to decision-making in industry and society.

### Relative share of GHG emissions in each life cycle stage

**Fig 3:**
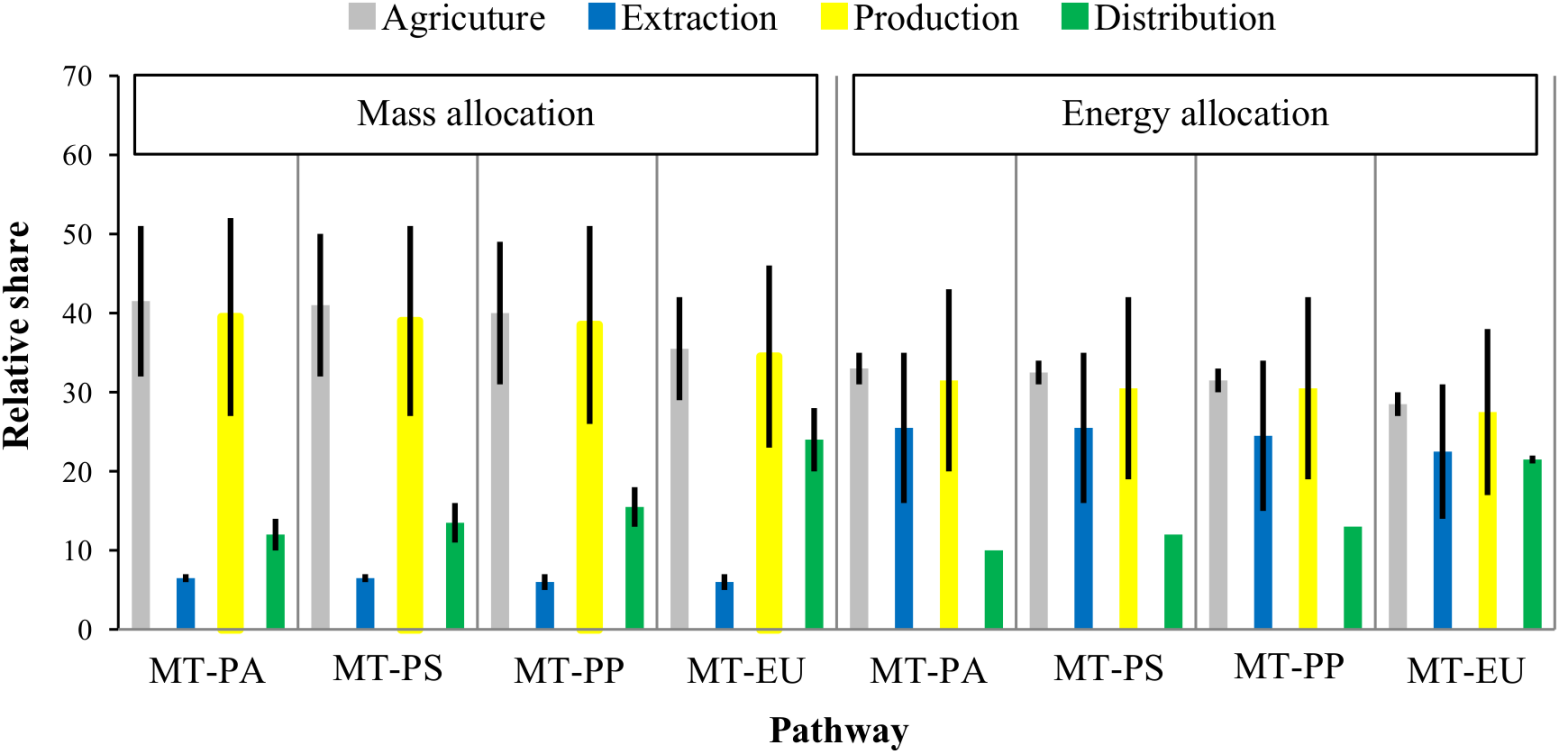
Relative share (allocation in mass and energy) of each stage (agriculture, extraction, biodiesel production and distribution) considering four scenarios for distribution of final B100.

Based on mass allocation (Fig 3) for all the pathways, the lowest relative share was observed in the extraction stage (MT-PA: 6 to 7%; MT-PS: 6 to 7%; MT-PP: 5 to 7%; MT-EU: 5 to 7%). Agricultural and biodiesel production stages have the highest emission shares for all four pathways, with roughly the same ranges in both stages (MT-PA: 32 to 50% and 27 to 51%; MT—PP: 32 to 51% and 27 to 52%; MT-PP: 31 to 49% and 26 to 51%; MT-EU: 29 to 42% and 23 to 46%).

By allocation of energy (Fig 3), the lowest relative share was observed in the distribution stage for three domestic pathways (MT-PS, MT-PA, MT-PP). Agricultural stage was still among the highest share for all pathways with little variation. Higher variation of relative range in the extraction and production stages makes them difficult to be compared with the agricultural stage. Domestic road transportation in all B100 products contributes 10 to 13% to the total life cycle GHG emissions. Similar result (13%) was observed by an environmental impact assessment of soybean supply chain from central-west region of Brazil to Europe [32]. In summary, the two stages with highest GHG emissions, both by using mass allocation and energy allocation, are agricultural and production stages (Fig 3). Extraction stage has the lowest GHG emissions by adopting mass allocation, while distribution stage inversely ranks the lowest by adopting energy allocation (*i*.*e*., high uncertainty exists in the extraction and distribution stages in the sensibility check).

### Potential GHG emissions from land use change (LUC) in Mato Grosso

Among all life cycle stages, GHG emissions from LUC are directly linked with the agricultural stage. Several studies have suggested LUC to be included in a life cycle GHG emission assessment [49–51] and studies with different scopes and methodologies including LUC did shown abruptly increased emissions (Table 1). Soybean expansion in Cerrado biome is no longer coupled with deforestation, but it is closely linked with LUC from extensive pastureland to agricultural use [52–53]. This “new” scenario of LUC leads to abrupt reduction of C debt associated to soybean production. Fargione et al.[49] suggested an annual repayment of 900 kg CO_2_ eq ha^-1^ y^-1^ carbon debt from Cerrado grassland to soybean field in 37 years payback time, whereas payback time from tropical rainforest to soybeans was estimated 319 years. Esteves et al.[51] applied a new approach to combine LUC with LCA in Mato Grosso do Sul and found that LUC converting pastures to soybean farming shared 81.2% of the annual emission increment (50.16 kg CO_2_ eq ha^-1^ y^-1^) in agricultural stage from 1993 to 2013. However, as it is also the case for Mato Grosso, a large share of degraded pasture exist in both of the two states, which is rather a source of emissions than an emission sink considered by IPCC. Besides, soybeans in Cerrado region (*i*.*e*., Mato Grosso) are mostly cultivated under no-tillage system, which makes it a carbon sink [54, 55]. In addition, inter-seasonal crops or adoption of crop-pasture rotation systems [55, 56] would also mitigate the impact from LUC. Therefore, to include LUC would lead our results of the agricultural stage to an abrupt change, but the changes could also vary drastically according to consideration of the LUC type, location, soil type and crop management. Thus, LUC was not included in this study, because we could not collect consistent data compatible with the quality and scale of our other input data. Nevertheless, it is of great importance for future studies to include LUC, or even indirect LUC (iLUC), *i*.*e*., change in the land use caused indirectly as a consequence of direct LUC taking place somewhere else in the world, to promote the optimal land use policy and to make soybean biodiesel a more sustainable renewable energy source.

### Domestic consumed B100 and exported B100

**Fig 4:**
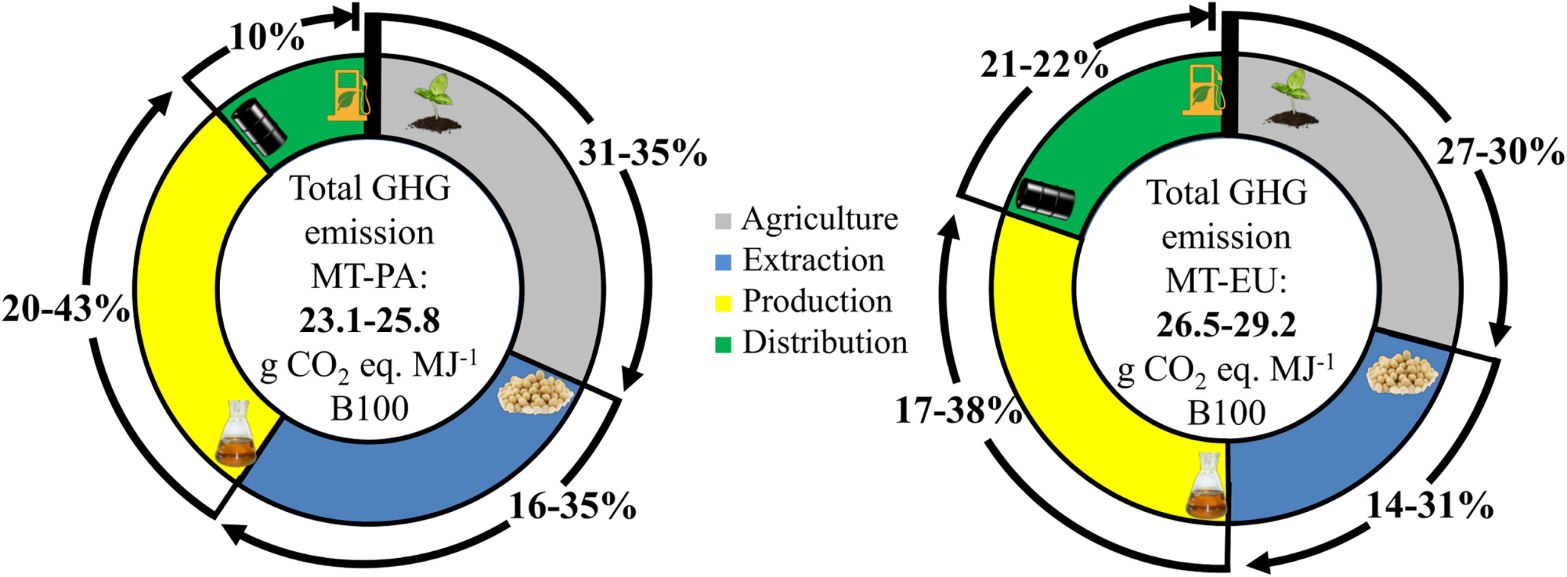
Life cycle GHG emission of biodiesel production, considering a domestic B100 scenario (MT-PA) and an exported B100 scenario (MT-EU).

A comparison of domestic consumed B100 (MT-PA pathway) and exported B100 (MT-EU pathway) scenarios, highlighting relative contribution of each stage, was provided in Fig 4. Life cycle GHG emissions for exported B100 to Europe was 12 to 13% higher (from 26.5 to 29.2 gCO_2_eq. MJ^-1^ B100) than that of domestic B100 (from 23.1 to 25.8 gCO_2_eq. MJ^-1^ B100). The factor associated to this increase was the long distance ocean shipping (transportation).

As Brazilian soybean prevails in EU countries [16, 20], the soybean biodiesel in Brazil based on four pathways has shown significant reduction in GHG emissions compare to the European diesel [9]. According to the EU Directive on Renewable Energy Sources (2009/28/EC), 35% of reduction in GHG emissions for biodiesel compared to fossil fuels is mandatory. Our findings, based on the unit of gCO_2_eq. MJ^-1^ B100, showed that B100 delivered to PA, PS, PP and EU has reduced 68 to 72%, 69 to 72%, 69 to 72% and 65 to 68% of GHG emissions compared to European fossil diesel, respectively. Regardless of the industrial plant considered, the calculation indicates a favorable condition for Brazilian soybean biodiesel, even considering the higher minimal reduction scheduled to be implemented in 2017 (50%) and in 2018 (60%). Compared to US fossil diesel, our finding on the domestic B100s also shows a reduction of 70–74% of GHG emissions, exceeding the minimal reduction threshold for biodiesel in the Renewable Fuel Standard [10].

**Fig 5:**
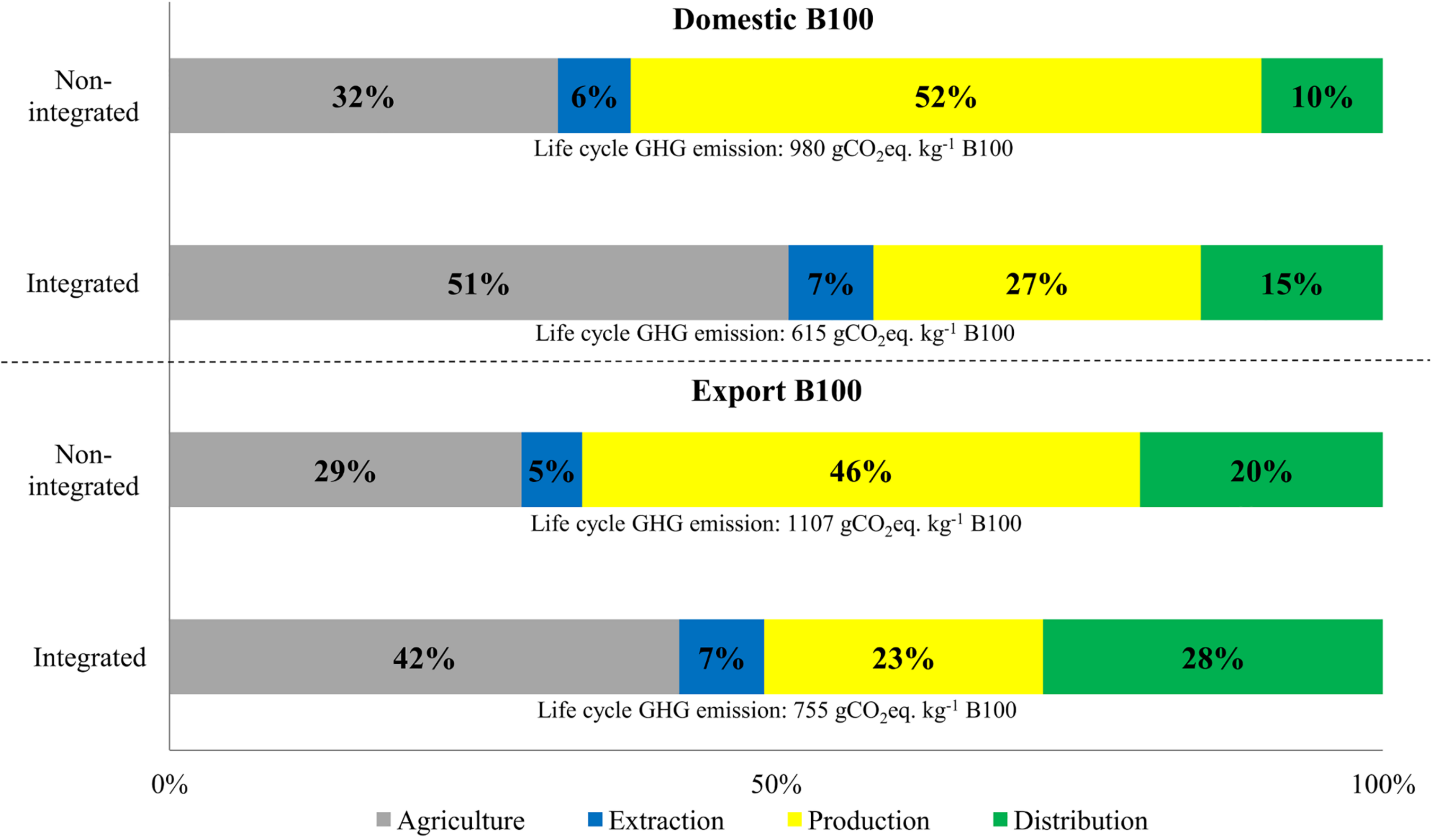
Relative share of GHG emissions in each stage (agriculture, extraction, biodiesel production and distribution) for B100 produced from integrated and non-integrated plant considering the MT-PA and MT-EU scenarios.

Fig 5 shows the comparison of MT-PA and MT-EU B100 produced in integrated system and non-integrated system. For both B100 products, integrated system significantly reduced the life cycle GHG emissions. Moreover, the relative share of emissions from production stage has been reduced more than half for both B100 products. Absence of transportation from extraction stage to B100 production stage (Fig 1), as well as, higher energy efficiency in an integrated system (industrial plant) are the primary drivers to decreasing total B100-associated GHG emissions (Fig 5). Therefore, it is essential to combine the efforts from government and industries to invest in integrated and more efficient biodiesel plants to make Brazilian B100 even more environmentally sustainable and commercially competitive. Our study did not mention the emissions from the final stage (*i*.*e*., combustion) of the life cycle, because the combustion of soybean biodiesel emits biogenic CO_2_, which is covered in the agriculture stage, and was considered as zero in this study.

As previously addressed, one shortage of this study is the exclusion of LUC in the calculation, which was due to a lack of sufficient data available for LUC calculation. Nevertheless, it is a pioneering study in Brazil to assess the life cycle GHG emissions of biodiesel with primary data from industry. We encourage future studies focused on LCA assessment to complement this study as well as research oriented to other feedstock sources (e.g., animal fat/wax, palm oil, sunflower among others) for biodiesel production in Brazil.

## Conclusions

This paper presents the life cycle GHG emission assessment of soybean biodiesel from the largest Brazilian soybean-producing region to four different destinations (MT-PA, MT-PS, MT-PP, MT-EU). Agricultural and biodiesel production stages were identified as the largest sources of GHG emissions, regardless of final B100 destination. Integrated system of industrial units for soybean production gains a significant improvement in reducing life cycle GHG emissions, for both soybean biodiesel and the by-products.

The life cycle GHG emissions for domestic B100 (MT-PA: from 23.1 to 25.8 gCO_2_eq. kg^-1^ B100) and exported B100 to Europe (MT-EU: from 26.5 to 29.2 gCO_2_eq. kg^-1^ B100) shows favorable conditions, in an environmental perspective, for Brazilian soybean biodiesel in the domestic market as well as international markets. Considering the development of agricultural techniques, logistic infrastructure, transportation network and even the emission factors specific to Brazilian biodiesel production, the GHG emissions from soybean biodiesel could be further optimized.

In compliance not only with the Brazilian National Program of Production and Use of Biodiesel to applying B8 (March 2017), B9 (March 2018) and B10 (March 2019) [13], but also with Brazil´s INDC presented in 2015 in Paris during UN/COP 21[57], this study suggests a high potential of soybean biodiesel to enhance the environmental sustainability of bio-based economy system in Brazil as well as in other biodiesel-importing countries.

## Supporting information

S1 Appendix Description of GHG emission calculation. (DOCX)
