# Supplementary material for: Assessing the greenhouse gas emissions of Brazilian soybean biodiesel production
Source: PLoS One. 2017 May 11;12(5):e0176948. doi: 10.1371/journal.pone.0176948 (PMC5426630; doi:10.1371/journal.pone.0176948)
Supplement: S1 Appendix — (DOCX) [file pone.0176948.s001.docx]

**Assessing the greenhouse gas emissions of Brazilian soybean biodiesel production**

Carlos E.P. Cerri, Xin You, Maurício R. Cherubin, Cindy S. Moreira, Guilherme S. Raucci, Bruno A. Castigioni, Priscila A. Alves, Domingos G.P. Cerri, Francisco F.C. Mello, Carlos C. Cerri

**Supporting Information**

**S1 Appendix. Descripion of GHG emission calculation**

The GHG-emission calculations were performed individually for each farm in the agriculture stage and for each industrial unit in the extraction and production stages as well as for each pathway in the distribution stage. Specific to the extraction and production stages, a general consideration of the difference between integrated and non-integrated production chain was adopted regardless of the individual efficiency of each working unit.

Emission factors used in this study is shown in Table S1. Two types of emission are generalized here: direct and upstream emissions. The direct emission type include all direct emissions occurred “in the gate”, from the source that are owned or controlled by the reporting entity, which is categorized in scope 1 by World Resource Institute (WRI, 2008). Upstream emission type in this study includes all the emission from the category - scope 2 and 3. Since downstream emissions are not included in this study, “Upstream” is the suitable term to represent emission by electricity purchasing in scope 2 as well as other indirect emissions in scope 3.

**Table S1.** List of emission factors used for calculation in this study

| Emission Source | Type of Emission | Unit | Value | Reference |
| --- | --- | --- | --- | --- |
| **Fuel** |  |  |  |  |
| Diesel | Upstream | kg CO_2_/ L diesel | 3,11 | Macedo et al., 2008 |
|  | Direct |  | 2,68 | IPCC, 2006 |
| Gasoline | Upstream | kg CO_2_/ L petrol | 2,85 | Macedo et al., 2008 |
|  | Direct |  | 2,33 | IPCC, 2006 |
| Ethanol | Upstream | kg CO_2_/ L ethanol | 0,46 | Macedo et al., 2008 |
|  | Direct |  | 0,01 | IPCC, 2006 |
| Biodiesel | Upstream | kg CO_2_/ L biodiesel | 0,39 | Almeida et al., 2008 |
|  | Direct |  | 0,01 | IPCC, 2006 |
| **Limestone** |  |  |  |  |
| Application in soil | Direct | kg CO_2_/ kg limestone | 0,48 | IPCC, 2006 |
| Production | Upstream |  | 0,01 | Ecoinvent, 2010 |
| Transportation | Upstream |  | 0,03 | Ecoinvent, 2010 |
| **Fertilizers** |  |  |  |  |
| Nitrogenous | Direct | kg N_2_O-N/ kg N | 0,01 | IPCC, 2006 |
| Urea | Direct | kg CO_2_/ kg urea | 0,73 | IPCC, 2006 |
| Nitrogenous | Upstream | kg CO_2_/ kg N | 3,14 | West & Marland, 2002 |
| Phosphate | Upstream | kg CO_2_/ kg P | 0,61 | West & Marland, 2002 |
| Potash | Upstream | kg CO_2_/ kg K | 0,44 | West & Marland, 2002 |
| **Electricity** |  |  |  |  |
| 2007-2008 | Upstream | ton CO_2_/ Megawatt/h | 0,04 | MCT, 2010 |
| 2008-2009 | Upstream |  | 0,03 | MCT, 2010 |
| 2009-2010 | Upstream |  | 0,03 | MCT, 2011 |
| **Farm chemicals** |  |  |  |  |
| Herbicides | Upstream | kg CO_2_ eq / kg I.A.  (Active Ingredient) | 17,24 | West & Marland, 2002 |
| Fungicides | Upstream |  | 18,98 | West & Marland, 2002 |
| Insecticides | Upstream |  | 18,08 | West & Marland, 2002 |
| **Seeds** |  |  |  |  |
| Soy seeds | Upstream | kg CO_2_ eq/ kg seed | 0,92 | West & Marland, 2002 |

For a better comparison and visualization of the results, the emissions of N_2_O and CH_4_ were converted to CO_2_ equivalent (CO_2_ eq). These conversions are necessary, because the GHG global warming potential of N_2_O (298) and CH_4_ (25) are different and significantly higher than that of CO_2_. The equations for calculation are as below:

**CO_2_ eq (N_2_O) = N_2_O * (44/28) * 298**

**CO_2_ eq (CH_4_) = C-CH_4_ * (16/12) *25**

where:

N_2_O: the amount of N_2_O flow

C-CH_4_: the amount of CH_4_ flow

(44/28): ratio between the molecular weight of N_2_O and nitrogen

(16/12): ratio between the molecular weight of CH_4_ and carbon

298: global warming potential of N_2_O over CO_2_

25: global warming potential of CH_4_ over CO_2_

The equations used to calculate each of the aspect in Table 4 with respective emission factors are shown below:

### Fuels

Two type of emissions were taken into consideration for emissions from fuels: “upstream” (emissions from the production and transportation of fuels) and “direct” (emissions from fuel combustion). According to the BNBP program (see also 1.2), commercial diesel in Brazil is blending mandatory. The ratios for the studied years in this study are: 2% for 2007/2008, 3% for 2008/2009, and 5% for 2009/2010. Regarding to the consumption of ethanol from upstream, we assumed in this study that the ethanol to gasoline ratio is 25% to 75%.

The equation for “upstream” emission calculation regarding fuels is shown below:

**F_COMB1_ = Tot x EF**

where,

F_COMB1_: upstream emission in CO_2_ eq ;

Tot: total amount of fuel used in respective stage (L);

EF: emission factor for each fuel used under “upstream” category (see also Table 4).

The equation for “direct” emission calculation regarding fuels is shown below:

**F_COMB2_ = Tot x EF**

where,

F_COMB2_: “direct” emission (combustion of fuels) in CO_2_ eq ;

Tot: total amount of fuel used in respective stage (L);

EF: emission factor for each fuel used under “direct” category (Table 4).

### Fertilizers

The direct GHG emissions in the agricultural stage are a result from the application of nitrogen fertilizers in the soil as well as the use of urea; Upstream emission in this context is the emission from producing each nutrient (N, P and K) for the formulation of synthetic fertilizers used in the agricultural stage.

The equation to calculate the upstream emission from synthetic fertilizers is:

**F_SN1_= A_TOT X_ Tot x %_EL_ x EF**

where,

F_SN1_: emissions from the producing of synthetic fertilized in CO_2_ eq;

A_TOT_ : Total area of soybean field (ha);

Tot : amount of synthetic fertilizer applied (kg fertilizer/ha);

%_EL_: percentage of the respective nutrient in the formulation (N, P and K);

EF : emission factors of respective nutrient (Table 4).

The equation for calculating direct emissions from nitrogen fertilizers is:

**F_SN2_= A_TOT X_ Tot x %_EL_ x EF**

where,

F_SN2_: emissions from the application of nitrogen fertilizer or urea in CO_2_ eq;

A_TOT_ : total area of soybean field (ha);

Tot : amount of (nitrogen) fertilizer applied (kg N/ha);

%_EL_: percentage of the respective nutrient in the formulation (N, P and K);

EF : emission factors of nitrogen fertilizers (Table 4).

### Limestone

The direct emission from the application of limestone as well as the emission from producing and transporting the product were considered in this category.

The equation for calculating upstream GHG emission from limestone application is:

**F_CAL1_ = A_TOT X_ Tot x EF**

where,

F_CAL1_ : emissions from the transport of limestone in CO_2_ eq;

A_TOT_ : total area of soybean field (ha);

Tot : amount of limestone applied in soil (kg /ha);

EF: emission factor in respective category (Table 4).

The equation for calculating direct GHG emission from limestone application is:

**F_CAL2_ = A_TOT X_ Tot x EF**

where,

F_CAL2_ : emissions from the transport of limestone in CO_2_ eq;

A_TOT_ : total area of soybean field (ha);

Tot : amount of limestone applied in soil (kg /ha);

EF: emission factor in respect category (Table 4).

### Farm chemicals

Only upstream emissions from the use of farm chemicals were calculated using specific emission factors for each chemicals (Ecoinvent, 2010). When specific emission factors cannot be found, reconsidering the active ingredient / the chemical group / the IUPAC (the International Union of Pure and Applied Chemistry) name, and a general emission factor can be applied under certain category (e.g. herbicides, insecticides or fungicides).

The equation for calculating upstream emissions from farm chemicals is:

**F_DEF_ = A_TOT X_ Tot x %_IA_ x EF**

where,

F_DEF_ : emissions from transporting respective farm chemicals in CO_2_ eq;

A_TOT_ : total area of soybean field (ha) (ha);

Tot : amount of chemicals applied(kg/ha);

%_IA_ : percentage of active ingredient in each chemical used (kg /L);

EF : emission factor under specific category (Table 4).

### Seeds

Only upstream emissions from producing the seed used in agricultural stage were considered. The equation for calculation is:

**F_SEM_ = A_TOT X_ Tot** **x EF**

where,

F_SEM_ : emission from production of the seed in CO_2_ eq;

A_TOT_ : total area of soybean field (ha) (ha);

Tot : amount of seed applied in the field (kg /ha);

EF : emission factor under specific category (Table 4).

### Residues

Emissions from field residues are calculated according to the recommendation of Brazilian National Communication (MCT, 2004). Total dry matter weight of soybean crops is assumed to be twice the total grain yield.

The equation for calculating emissions from field residues is:

**F_CR_= 2*P_TOT_ x N_SOJ_ x (1-F_REMOV_) x EF**

where,

F_CR_ : emissions from decomposition of field residues in CO_2_ eq;

P_TOT_ : total soybean yield (kg);

N_SOJ_ : total N content in soybean crop in kg N/ kg dry matter; it was assumed to be 3% according to MCT (2004);

F_REMOV_ : percentage of the total crop biomass removed from the field compared to total biomass harvested; it was assumed to be 45% according to MCT (2004).

EF : emission factor under specific category (Table 4).

### Electricity

The emissions from the use of electricity are calculated as an upstream emission. The equation for calculation is:

**F_ENE_ = Tot x EF**

where,

F_ENE_ = emission from using electricity;

Tot = total energy consumed (MW/h)

EF = emission factor under specific category (Table 4).

Based on above descript calculation element, the system description in 2.3 and listed emission factors in 2.4, the calculation for each stage can be summarized as:

F = ∑ {step1 (upstream + direct emissions); step2 (upstream + direct emissions), …}

where,

steps are shown in the system description (Figure 3) with emission arrow “↑”;

upstream and direct emissions are calculated based on above categories.

## Allocation of emissions

The international standards ISO 14040 and ISO 14044 were used to guide the allocation criteria. The following criteria were adopted:

- Whenever possible, allocation of emissions was avoided, and a separate analysis made of the production systems for the various products produced in the same area;
- When a separate analysis of the inputs for each crop was not possible, such as the application of lime to the soil, the electricity consumed on the farm, among others, the allocation criterion was based on the production area of each crop in the same agricultural year.

Mass allocation was chosen for this study, which is commonly used in LCA studies, because it is easy to be applied and provides reasonable results (Vigon et al., 1993). In this study, we observed production data from both integrated and non-integrated production chain to determine the conversion factors. The allocation factor for oil (≈20% for mass allocation; 36% for energy allocation) and meal (≈80% for mass allocation; 63% for energy allocation) was based on the production data from refinery (Table S2). Also, mass allocation of 90% biodiesel to 10% glycerin was observed in non-integrated system and that in an integrated system was 94% biodiesel to 6% glycerin (Table S2). Energy allocation between biodiesel (94%) and glycerin (6%) was based on reference value in the EU RED.

**Table S2**. Production data from refinery (integrated and non-integrated) in the year 2008/2009

|  | Input (soybean) | Soybean meal | Crude oil |
| --- | --- | --- | --- |
|  | Extraction Stage | | |
| Mass (t) | 1,663,308.06 | 1,400,718.22 | 345,932 |
| Percentage (%) |  | ≈ 80 | ≈ 20 |
| Energy (MJ) | 65,905,255,261 | 24,172,334,350 | 13,640,087,509 |
| Percentage (%) |  | 64 | 36 |
|  | Input (crude oil) | Biodiesel | Glycerin |
|  | Production Stage (non-integrated system) | | |
| Mass (t) | 13,216 | 11,935 | 1,326 |
| Percentage (%) |  | 90 | 10 |
|  | Production Stage (integrated system) | | |
| Mass (t) | 152,466 | 150,009 | 18,996 |
| Percentage (%) |  | 94 | 6 |

Accompanying energy allocation was used in accordance with the EU RED for comparison and discussion as well as to test the sensitivity of the results. Allocation of economic value was not applied in this study. Not only because it is not the scope of this study but also because the economic value of soy oil and soy meal are volatile, requiring data to be updated frequently. Specific to Brazil, the exchange rate of Brazilian currency fluctuated dramatically, which made it even harder to evaluate the market value in a global context.

The life cycle GHG emission analyses of final B100 product based on four different routes (MT-PA, MT-PS, MT-PP, MT-EU) were performed and the environmental implications are discussed based on the EU Directive on Renewable Energy Source (2009/28/EC). Finally, a comparison of life cycle GHG emission of domestic and export B100; and the performances of integrated and non-integrated system were also presented.

References

Almeida, M.C., Alves, R.P., Velasquez, S.M.S.G., Coelho, S.T., Moreira, J.R. Externalidades e análise do ciclo de vida do biodiesel. CENBIO – Centro Nacional de Referencia em Biomassa. 2008, 160 p.

Ecoinvent. Life-cycle inventory data base. “Swiss Centre for Life Cycle Inventories of the Swiss Federal Institute of Technology, Zurich.” Accessed July 04. http://www.ecoinvent.org

IPCC – Intergovernmental Panel on Climate Change, ed. 2006. Guidelines for National Greenhouse Gas Inventories. Hayama, Japan: National Greenhouse Gas Inventories Programme, Institute for Global Environmental Strategies.

ISO. 2006. ISO 14044: Environmental Management, Life Cycle Assessment, Requirements and Guidelines. International Organization for Standardization.

Macedo I.C., Seabra J.E.A. and Silva J.E. 2008. “Green house gases emissions in the production and use of ethanol from sugarcane in Brazil: The 2005/2006 averages and a prediction for 2020.” Biomass and bioenergy, 32 7: 582–95.

MCT - Ministério da Ciência e Tecnologia. Primeiro Inventário Brasileiro de Emissões Antrópicas de Gases de Efeito Estufa. Relatórios de Referência. Emissões de Gases de Efeito Estufa por Fontes Móveis, no Setor Energético, 2004.

MCT - Ministério da Ciência e Tecnologia. Fatores de Emissão de CO2 para utilizações que necessitam do fator médio de emissão do Sistema Interligado Nacional do Brasil, como, por exemplo, inventários corporativos, 2010.

Vigon B.W. and Harrison C.L. 1993. “Life-cycle assessment: Inventory guidelines and principles.”.

West, T., Marland, G. A synthesis of carbon sequestration, carbon emissions, and net carbon flux in agriculture: comparing tillage practices in the United States. Agriculture, Ecosystems and Environment, v. 91, p. 217-232, 2002.

WRI - World Resources Institute. 2015. “A closer look at Brazil’s new climate plan (INDC).” Accessed 08.18.2016. http://www.wri.org/closer-look-brazils-new-climate-plan-indc
